# Effects of aqueous suppressants and prostaglandin analogues on early wound healing after glaucoma implant surgery

**DOI:** 10.1038/s41598-019-41790-1

**Published:** 2019-03-27

**Authors:** Kyoung In Jung, Jung Eun Woo, Chan Kee Park

**Affiliations:** Department of Ophthalmology, Seoul St. Mary’s Hospital, College of Medicine, The Catholic University of Korea, Seoul, Republic of Korea

## Abstract

A hypertensive phase frequently develops in the early postoperative period after glaucoma shunt operations. Anti-glaucoma eye drop use is essential when postoperative intraocular pressure (IOP) is not controlled. We investigated whether the use of early topical anti-glaucoma medication affects wound healing following glaucoma tube surgery. Eyes were randomly assigned to receive topical aqueous suppressant (timolol-dorzolamide fixed combination), prostaglandin (PG) analogue (travoprost), or normal saline (control group). First, we observed the effects of topical eye drops on Tenon’s tissue in non-operated eyes in rabbits. Second, we examined the effects of these eye drops on rabbit eyes that underwent Ahmed glaucoma drainage device implantation, including the effects on the histopathological appearance of their blebs. Interleukin-2 in the Tenon’s tissue was elevated in the PG group when compared to the control and aqueous suppressant groups (P = 0.006). In non-operated eyes, IOP was similar among the groups (P = 0.545). After glaucoma implant surgery, the average height of the inner collagenous layer and the average height of the α-SMA-positive blebs were the least in the aqueous suppressant group (P = 0.013, P = 0.001, respectively) at 4 weeks postoperatively. IOP was lower in the aqueous suppressant group than that in the control and PG groups (P = 0.001) following tube surgery. After Ahmed tube surgery, early treatment with aqueous suppressant decreased fibrosis in the bleb, but early treatment with the PG analogues did not.

## Introduction

In glaucoma, uncontrolled intraocular pressure (IOP) can cause loss of vision. Glaucoma surgery is generally needed to decrease IOP in glaucoma patients with medically uncontrolled IOP. Tube surgery has become a popular alternative to conventional filtration surgery for treating refractory glaucoma^1^. Medical claims data from the USA show a 410% increase in the use of glaucoma shunts between 1994 and 2012 and a concurrent 77% decrease in the use of trabeculectomy^1^. However, the surgical failure rate reached approximately 30% in the 5 years after tube surgery^2^. The hypertensive phase, a remarkable IOP elevation that occurs during the early postoperative phase (approximately 1 month), is problematic following shunt operation, especially after Ahmed glaucoma implant surgery^3–7^. We previously found that the bleb wall was the thinnest and the most hyper-reflective during the hypertensive phase using anterior-segment optical coherence tomography after Ahmed glaucoma valve implantation^3^. We assumed that the disappearance of oedema and the aggravation of fibrosis, wound contraction, or cross-linking of collagen, could be the cause of the hypertensive phase, but the exact mechanism of the hypertensive phase is not completely understood^3,6,8^.

Recently, early aqueous suppressant treatment has improved the success rate and decreased the occurrence of the hypertensive phase after Ahmed glaucoma implantation^9,10^. Aqueous humour contains many growth factors, including transforming growth factor (TGF)-β, which is important in fibrosis during the wound healing response and foreign body reaction^11,12^. Previously, we demonstrated that fibrotic encapsulation increased more with aqueous humour flow than without aqueous humour flow in glaucoma drainage device surgery^13^. On the other hand, topical prostaglandin (PG) analogues are the agents most commonly used to reduce IOP and have become front-line treatments for glaucoma due to their once-daily dosing and high efficacy^14^. IOP reduction by PGF_2α_ is suspected to be induced by the relaxation of the ciliary muscle and modification of its extracellular matrix (ECM)^15^. PGF_2α_ is also involved in acute and chronic inflammatory disease^16^. In one *in vitro* study, latanoprost induced collagen gel contraction^17^. Given those findings, the use of aqueous suppressants or prostaglandin analogues early after glaucoma drainage device (GDD) surgery might also affect the wound healing process.

In glaucoma implant surgery, we observed a densely arranged collagenous layer in the capsule^13^. Following glaucoma shunt surgery, IOP reduction depends on the degree of fibrotic encapsulation around the endplate^18,19^. During the hypertensive phase after implant surgery, the use of anti-glaucoma eye drops is inevitable. The appropriate selection of anti-glaucoma eye drops after glaucoma shunt operations can be important for the wound healing response during the early postoperative period. However, no histological study has explored whether aqueous suppressants and PG analogues affect the wound healing process following glaucoma shunt implantation. First, we analysed the effects of aqueous suppressants and PG analogues on inflammatory and fibrosis-related cytokines in aqueous and Tenon’s tissue in a naïve animal model. Second, we observed the effects of aqueous suppressants and PG analogue anti-glaucoma eye drops on wound healing after GDD implantation in an experimental model *in vivo*.

## Methods

Adult New Zealand white rabbits (2–3 kg, 20 weeks old) were used in accordance with the ARVO Statement on the Use of Animals in Ophthalmic and Vision Research. All processes were allowed by the Institutional Animal Care and Use Committee of the School of Medicine, Catholic University of Korea.

Aqueous suppressant group eyes received topical preservative-free timolol (0.5%)-dorzolamide (0.2%) fixed-combination drops (Cosopt-S^®^, Santen OY, Tampere, Finland) twice daily. The eyes in the PG group received 0.004% travoprost preserved with Polyquad (Travatan^®^, Alcon Laboratories, Texas, USA) once daily. Control group eyes received normal saline. All rabbits were treated with eye drops or normal saline for 4 weeks.

IOP was assessed under general anaesthesia at baseline and 4 weeks postoperatively using a rebound tonometer (TonoVet®). IOP was measured 5 times. Mean IOP measurements were compared among the groups.

### Non-glaucoma aqueous shunt model

Right or left eyes were randomly assigned to the aqueous suppressant, PG, or control group.

Aqueous humour and a 4 × 5-mm piece of Tenon’s tissue were collected from rabbits 4 weeks after the application of glaucoma medication or normal saline (~150 μL). Each sample was immediately frozen and stored at −70 °C until analysis. The Tenon’s tissue samples were cut into small pieces and placed in radioimmunoprecipitation assay buffer. The proteins were extracted using a lysis kit (hard tissue homogenizing kit CK28, Precellys) according to the manufacturer’s instructions. The tissue extracts were incubated on ice (10 min) and centrifuged (14,000 g, 25 min, 4 °C). Total protein in the Tenon’s tissue extracts was quantitatively analysed using a standard BCA assay (Pierce, Rockford, IL, USA).

Multiple cytokines were simultaneously analysed in the aqueous humour and Tenon’s tissue samples using a bead-based multiplex cytokine assay (XMAP; Luminex Corp., Austin, TX, USA). Using assay kits (LXSAHM; R&D systems), interleukin (IL)-2, IL-6, macrophage chemotactic protein-1 (MCP-1), and vascular endothelial growth factor (VEGF) levels were determined. TGF β-1, -2, and -3 levels were analysed using a TGFBMAG assay kit (Merck Millipore). Matrix metalloproteinase (MMP)-3 and -9 and tissue inhibitor of metalloproteinase (TIMP)-2 and -4 levels were analysed in the Tenon’s tissue samples using a bead-based multiplex cytokine assay kit (xMAgLxSA for MMP, hTIMPMag kit for TIMP; both from R&D systems).

### Glaucoma aqueous shunts model

GDD implantation was performed using Model FP 8 Ahmed glaucoma valves (New World Medical Inc., Rancho Cucamonga, CA, USA). Ahmed shunts were implanted in both eyes of each animal; each eye was randomly allocated to the aqueous suppressant group, PG group, or control group.

The operation was done under general anaesthesia, which was administered as an intramuscular injection of 15 mg/kg tiletamine plus zolazepam (Zoletil) and 5 mg/kg xylazine hydrochloride (Rompun). Local anaesthesia was achieved using proparacaine hydrochloride (Alcaine 0.5%).

The details of this GDD implantation procedure have been reported previously^20^.

Briefly, a plate was fixed to the sclera after the creation of a fornix-based conjunctival flap. Tube priming was performed by irrigation with saline and was followed by insertion of the tube into the anterior chamber (AC). The tube was then loosely fixated to the sclera with a 10-0 nylon suture. The conjunctiva was reapproximated with 8-0 Vicryl sutures. Topical treatment (aqueous suppressant, PG analogues, or normal saline) started 1 week postoperatively.

The animals were euthanized by an intravenous administration of potassium chloride at 2 and 4 weeks postoperatively. All eyes underwent slit-lamp examination to identify the patent tube tip and to observe the bleb. After enucleation, all eyes were incubated overnight in 4% paraformaldehyde at room temperature.

### Histochemical staining

**Figure 1:**
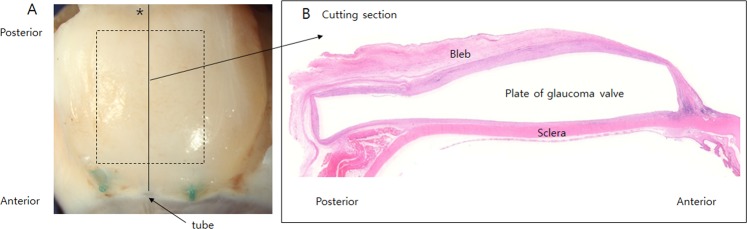
(**A**) The bleb was cut over the endplate along the midline (asterisk) corresponding to the axis of the tube. (**B**) The cross section at the midline of the capsule was analysed.

After fixation, the bleb was severed over the endplate along the line of the tube for histological examination (Fig. 1)^20^. Sections on both sides along the midline were analysed. Ten 3-µm-thick tissue sections were obtained and embedded in paraffin before being stained using haematoxylin and eosin (H&E) or a Masson’s trichrome staining kit (HT15; Sigma-Aldrich, St. Louis, MO, USA) to stain collagen. In H&E-stained slides, the number of foreign body giant cells were analysed after excluding the number of foreign body giant cells around the suture material.

### Immunohistochemistry

Immunohistochemical staining was performed using standard protocols and dextran polymer reagents (Envision + DAM system; Dako, Glostrup, Denmark). For antigen retrieval, the sections were incubated for 20 min at 95 °C in Target Retrieval Solution, Citrate pH 6 (Dako), using PTLink (Dako). The slides were immersed first in peroxidase blocking solution (Dako) for 10 min and then in monoclonal anti α-smooth muscle actin (SMA) (1:4500; Sigma-Aldrich). Antibodies were detected using the dextran polymer reagent for mouse antibody for 30 min. The slides were stained with 3,3′-diaminobenzidine (DAB) for 10 min, and the nuclei were stained with Meyer’s haematoxylin for 2 min. Sections were mounted onto slides (Permount; Fischer Scientific, Pittsburgh, PA, USA).

The slides were observed using a microscope (Nikon, Tokyo, Japan) and scanned using a panoramic MIDI scanner (3DHISTECH Ltd., Budapest, Hungary). The images produced were analysed using the image analysis algorithms included with the instrument. The thickness of the collagen-rich innermost layer in the bleb was measured using the included distance-measuring tool in the device. Five height readings along the capsule were averaged and compared between the three groups. The mean of five height values of the α-SMA immunostained bleb were measured using the tool included with the scanner.

### Statistical analysis

Statistical analyses were performed using SPSS software, version 18.0 (SPSS Inc., Chicago, IL, USA). Differences in parameters among the groups were analysed using ANOVA. *P* values less than 0.05 indicate significant differences.

## Results

Five animals were assigned to each treatment group. There were a total of 9 treatment groups across the study. For the non-operated eyes, three different treatment groups were used. For the operated eyes, there were also three different treatment groups each at postoperative 2 weeks and 4 weeks.

### Non-glaucoma aqueous shunt model

**Figure 2:**
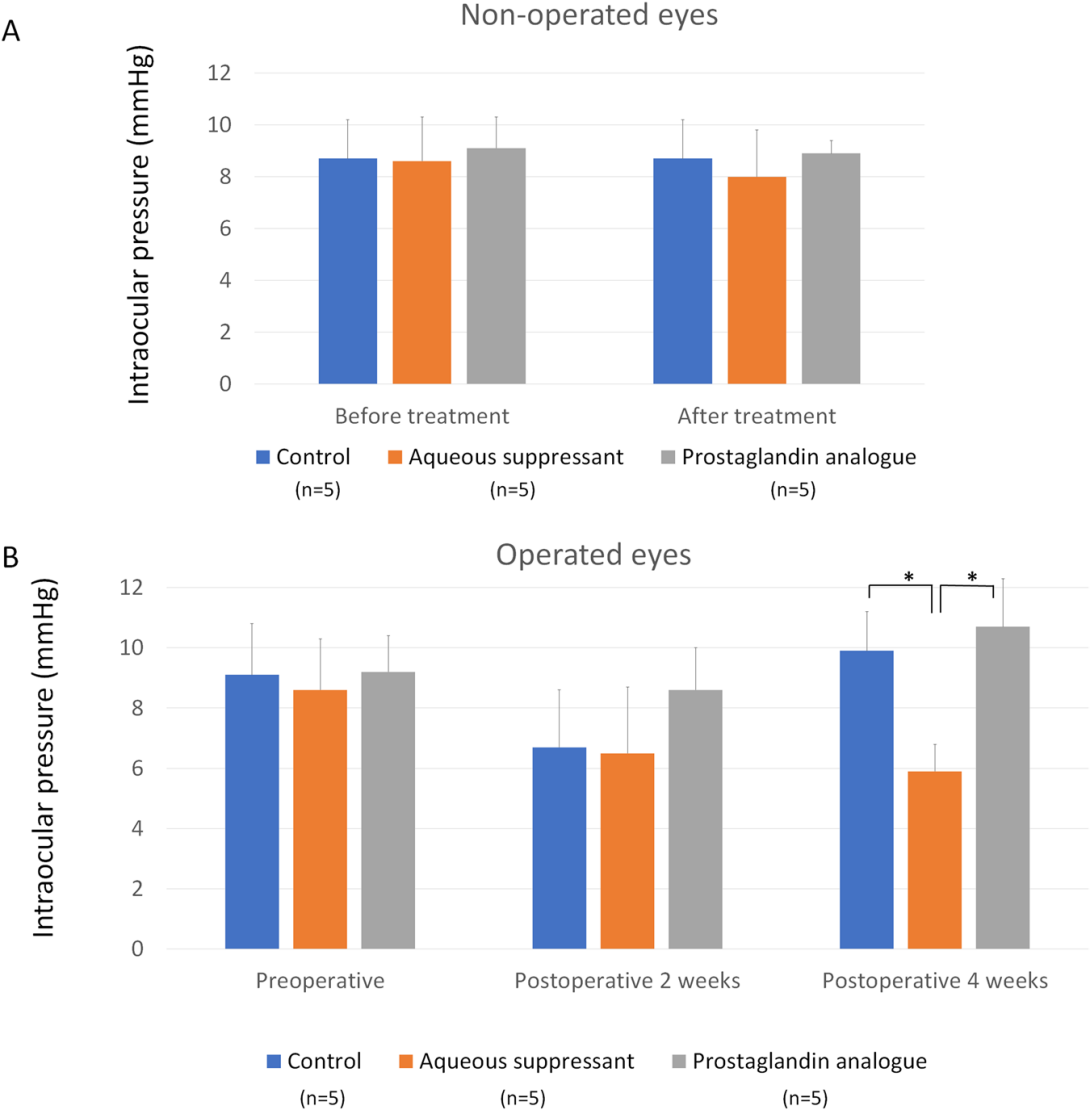
(**A**) Intraocular pressure (IOP) in non-operated eyes. IOP did not significantly differ at baseline and 4 weeks after treatment with glaucoma medication or normal saline among the control group, the aqueous suppressant group, and the prostaglandin (PG) group (P = 0.670, 0.545, respectively). (**B**) IOP during glaucoma drainage device implantation. In the operated eyes, preoperative IOP was similar among the groups (P = 0.200). Postoperative IOP was lower in the aqueous suppressant group than in the control or PG group (P = 0.001).

In the non-operated eyes, baseline IOP was not significantly different among the groups (P = 0.670). IOP was 8.7 ± 1.5 mmHg in the control group, 8.0 ± 1.8 mmHg in the aqueous suppressant group, and 8.9 ± 0.5 mmHg in the PG group. Measurements were taken 4 weeks after treatment with glaucoma medication or saline (P = 0.545; Fig. 2, Supplementary Table S1).

**Figure 3:**
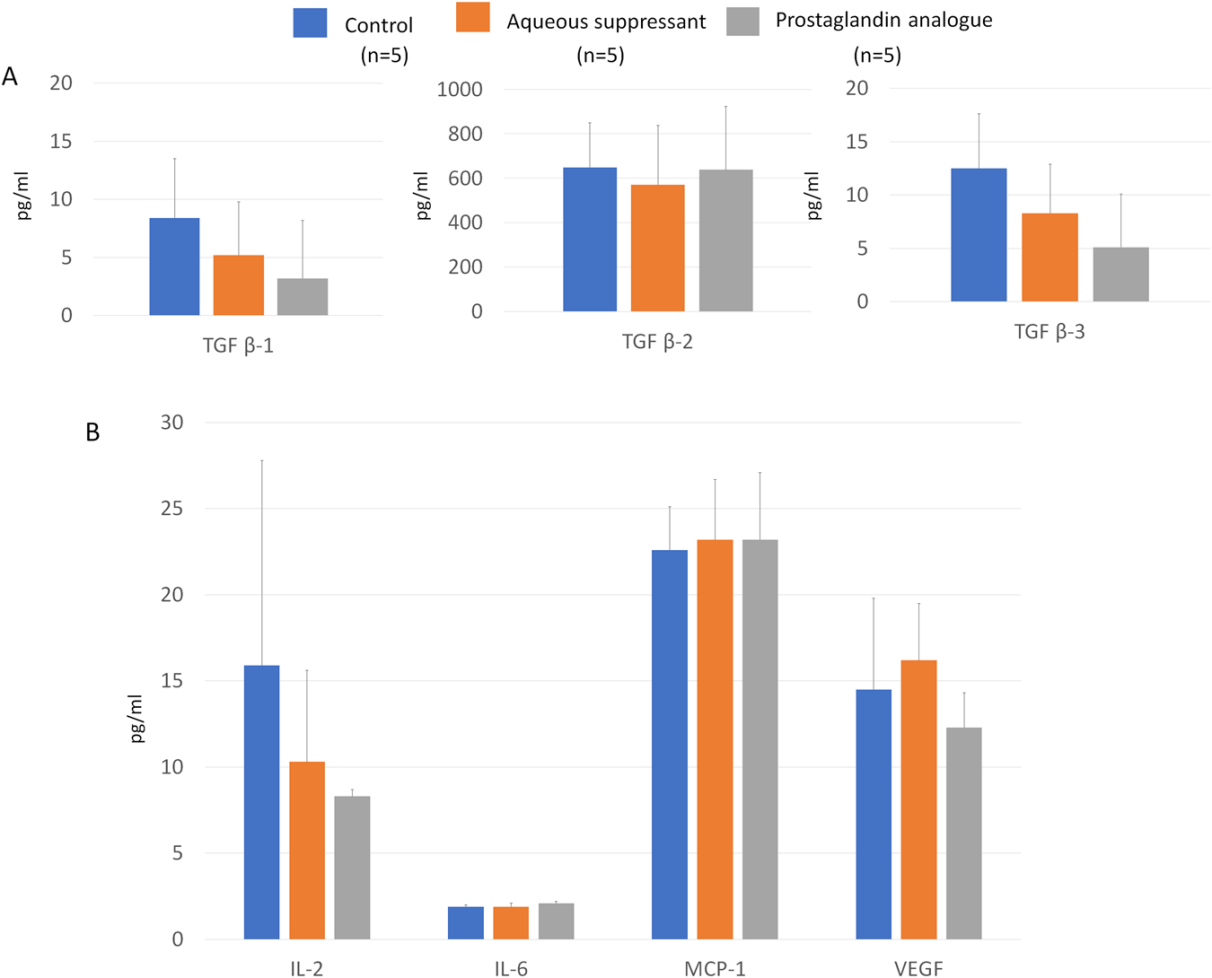
Cytokine levels of the aqueous humour of non-operated eyes. (**A**) Transforming growth factor (TGF) β-1, -2, and -3 levels, (**B**) Interleukin (IL)-2, IL-6, macrophage chemotactic protein-1 (MCP-1), and vascular endothelial growth factor (VEGF) did not significantly differ between the aqueous suppressant, prostaglandin analogue, and normal saline treatment groups (all P > 0.05).

In the aqueous humour, we found no significant differences in TGF β-1, -2, -3, IL-2, -6, MCP-1, and VEGF levels among the three groups (P > 0.05; Fig. 3, Supplementary Table S2).

**Figure 4:**
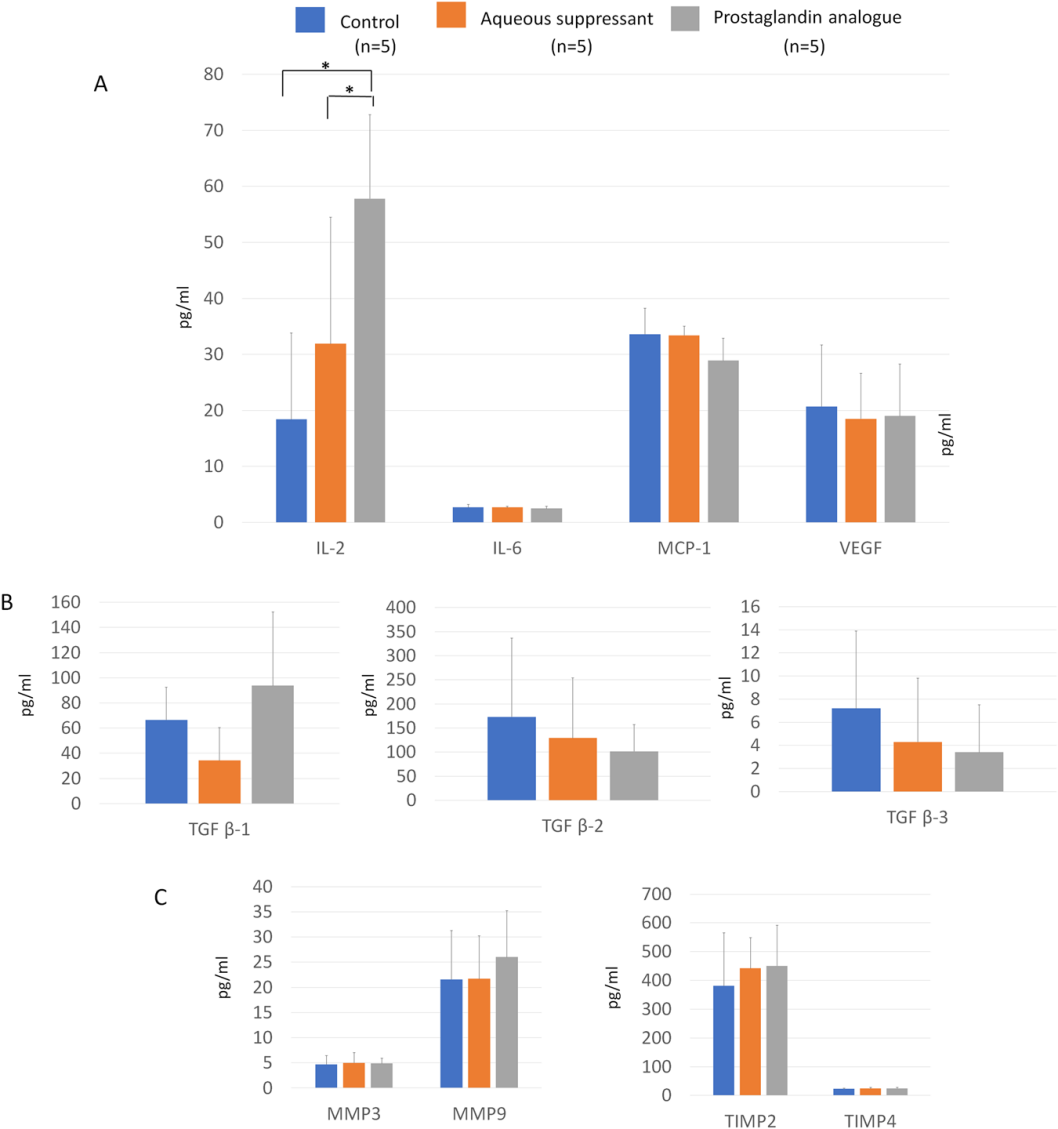
Cytokine levels of Tenon’s tissue in non-operated eyes. (**A**) Interleukin (IL)-2 level was higher in the prostaglandin (PG) group than in the control and aqueous suppressant groups (P = 0.006). (**A**) IL-6, macrophage chemotactic protein-1 (MCP-1), vascular endothelial growth factor (VEGF), (**B**) transforming growth factor (TGF) β-1, -2, and -3, (**C**) matrix metalloproteinase (MMP)-3 and -9 and tissue inhibitor of metalloproteinase (TIMP)-2 and -4 levels did not vary among the three groups (all P > 0.05).

For Tenon’s tissue, IL-2 levels were higher in the PG group than in the control or aqueous suppressant group (P = 0.006). TGF β-1, -2, -3, IL-6, MCP-1, and VEGF did not significantly differ among the groups (All P > 0.05; Fig. 4, Supplementary Table S3). There was no significant difference in MMP-3 or -9 levels among the groups (All P > 0.05).

### Glaucoma aqueous shunt model

After GDD implantation, all rabbits appeared free from systemic complications.

For the operated eyes, preoperative IOP was 9.1 ± 1.7 mmHg in the control group, 8.6 ± 1.7 mmHg in the aqueous suppressant group, and 9.1 ± 1.2 mmHg in the PG group (*P* = 0.200). IOP at 4 weeks postoperatively was 9.9 ± 1.3 mmHg in the control group, 5.9 ± 0.9 mmHg in the aqueous suppressant group, and 10.7 ± 1.6 mmHg in the PG group (*P* = 0.001; Fig. 2, Supplementary Table S1).

### Histochemical staining

**Figure 5:**
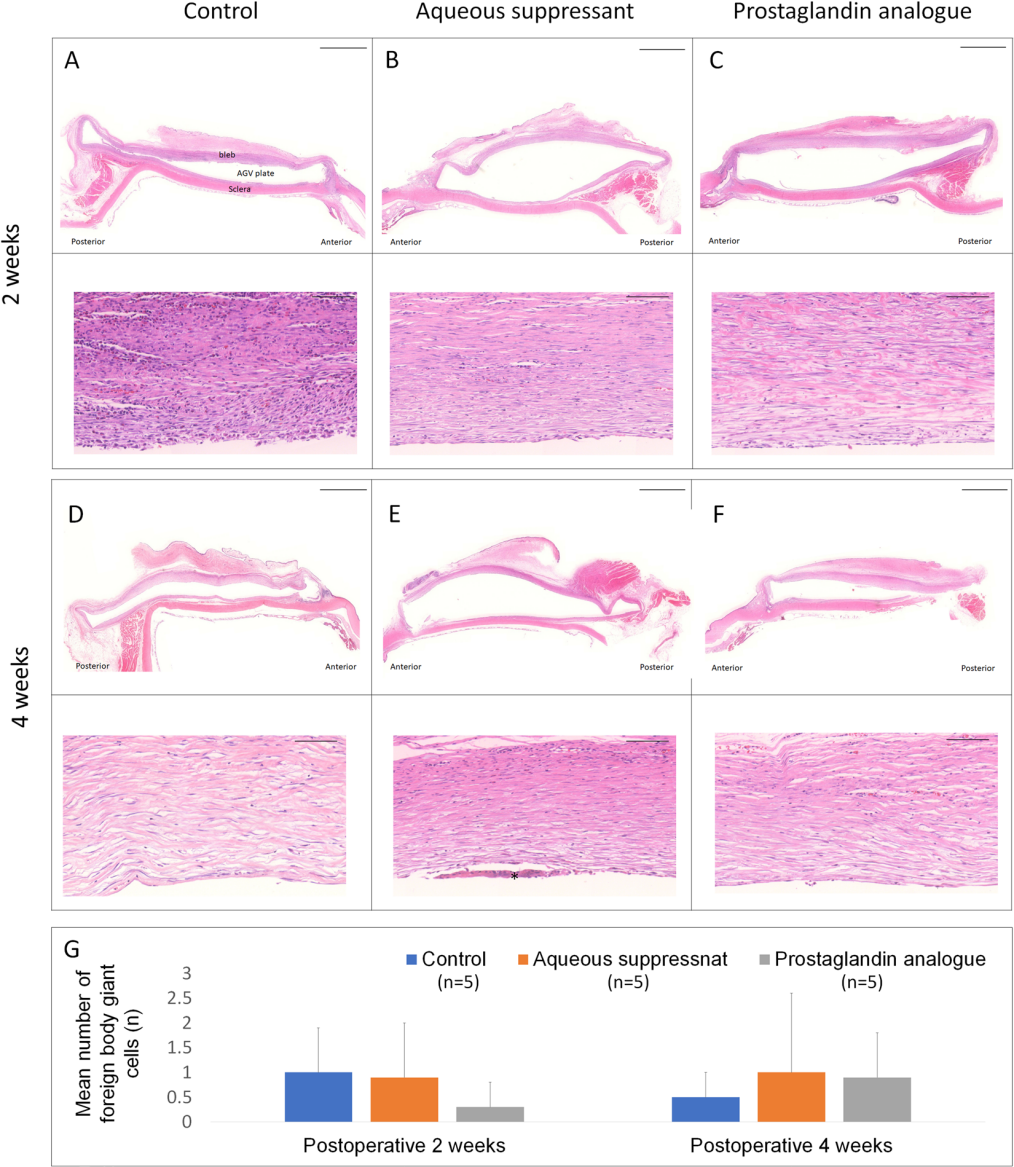
Histological micrographs of the capsules surrounding the endplate after H&E staining. Cellularity was higher at 2 weeks postoperatively than at 4 weeks postoperatively. Generally, the degree of cellularity was lower in the aqueous suppressant group than in the control or prostaglandin group, but the difference among the groups was not statistically significant (P > 0.05). The mean number of foreign body giant cells (asterisk) per slide was similar among the groups (P > 0.05). Scale bar: 2 mm in upper rows (**A**–**F**), 0.1 mm in lower rows.

Two weeks postoperatively, prominent fibroblast cells and a few inflammatory cells were found in the bleb. The cellular density was higher in the inner bleb area than in the outer bleb area (Fig. 5, Supplementary Table S4). The cellularity decreased from 2 to 4 weeks postoperatively. The cellularity was not significantly different among the groups at 2 (P = 0.556) and 4 (P = 0.631) weeks postoperatively. The number of foreign body giant cells was similar among the groups 2 weeks postoperatively (P = 0.254) and 4 weeks postoperatively (P = 0.716).

**Figure 6:**
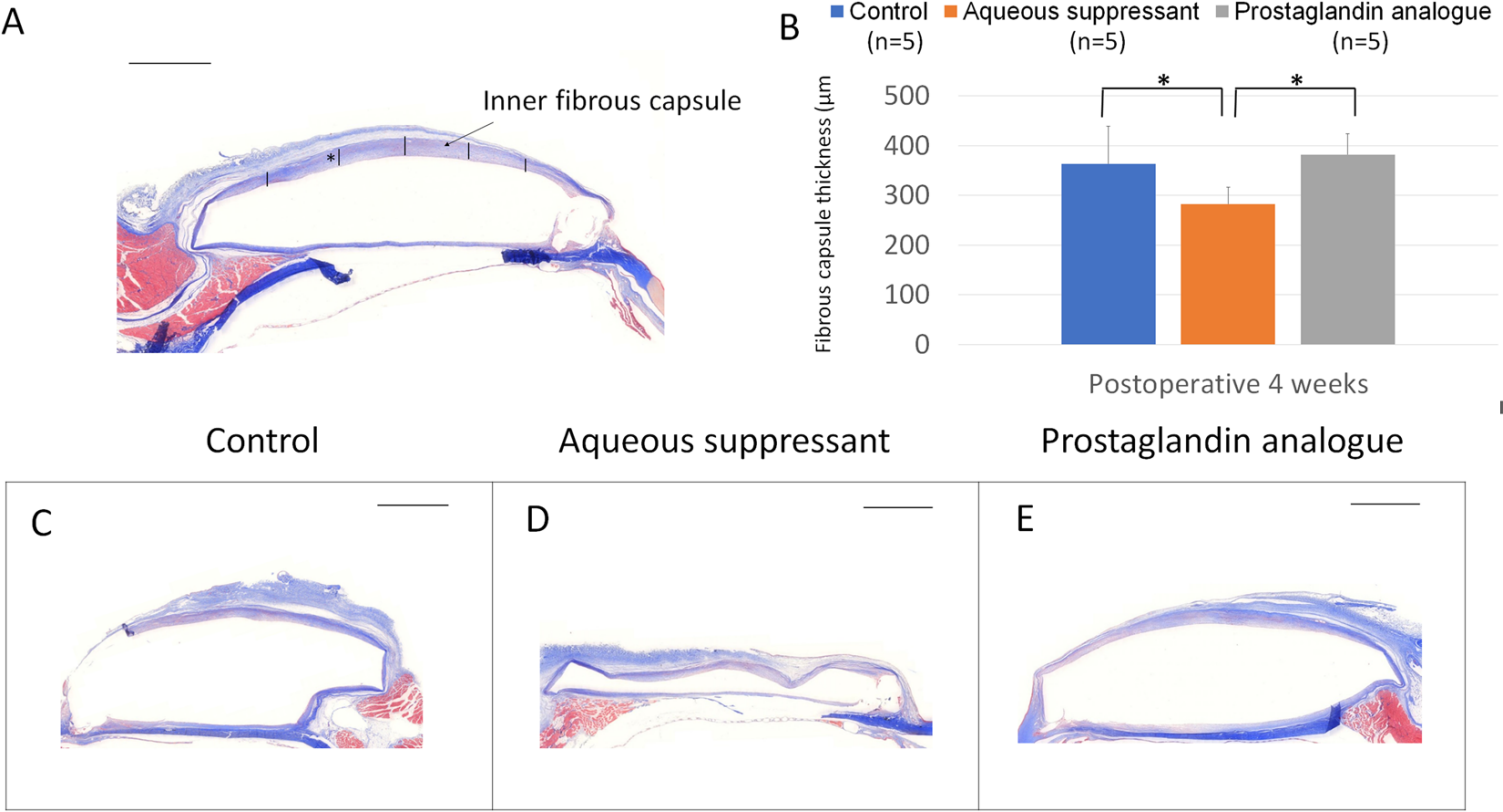
Collagen deposition stained by Masson trichrome staining. (**A**) The height of the inner fibrous capsule in the bleb was measured. (**B**,**C**) The mean height of the inner fibrous capsule was smaller in the aqueous suppressant group than in the control or prostaglandin group (P = 0.013). Scale bar: 2 mm.

Masson’s trichrome staining revealed that the bleb was stained moderately blue at 4 weeks postoperatively. The inner bleb was well-preserved relative to the outer bleb during tissue preparation and staining. We quantitatively analysed the height of the inner collagenous layer (Fig. 6, Supplementary Table S5). The mean of five height readings from the inner bleb was lower in the aqueous suppressant group than in the control or PG group (P = 0.013).

### Immunohistochemistry

**Figure 7:**
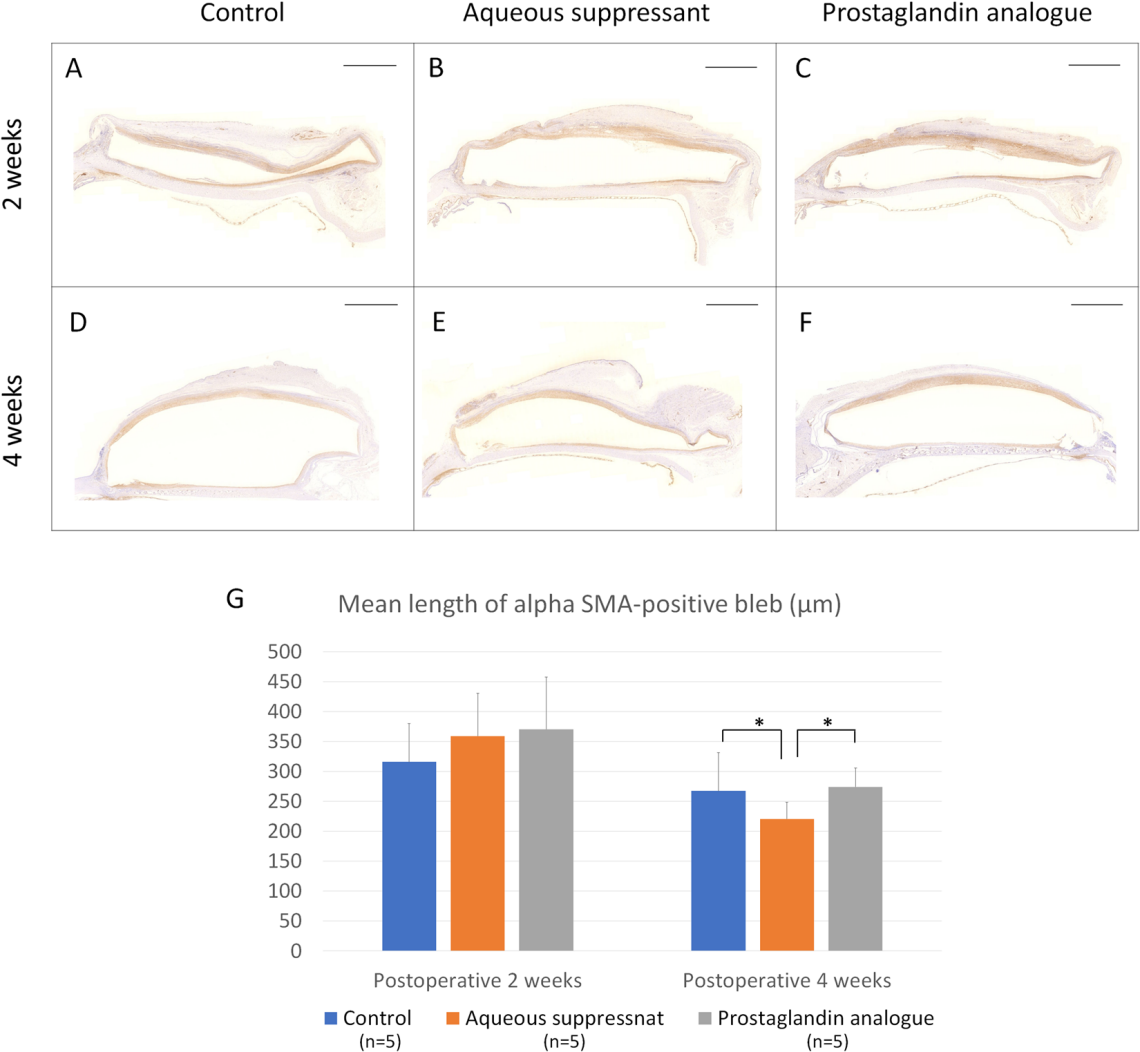
Transformation of fibroblasts to myofibroblasts. (**A**,**B**) Anti α-smooth muscle actin (SMA) immunohistochemical staining did not reveal a significant difference among the three groups at postoperative 2 weeks. At postoperative 4 weeks, the mean length of the α-SMA positive bleb was lower in the aqueous suppressant group than in the control or PG group (P = 0.001). Scale bar: 2 mm.

Expression of α-SMA was prominent in the inner bleb area at both 2 and 4 weeks postoperatively (Fig. 7, Supplementary Table S6). Two weeks postoperatively, the average height of the α-SMA-positive inner bleb was similar among the three groups (P = 0.100). At 4 weeks postoperatively, the average height of the α-SMA-positive blebs was lowest in the aqueous suppressant groups (*P* = 0.001).

**Figure 8:**
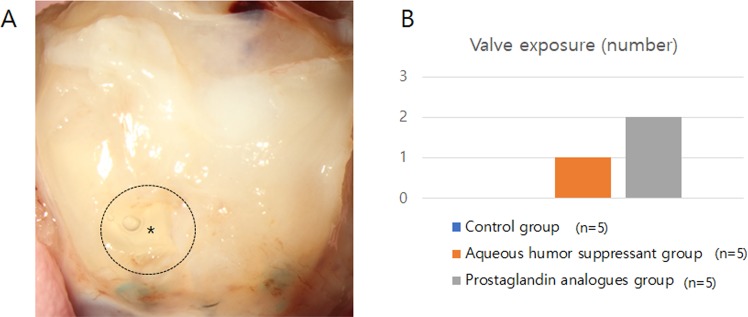
Valve exposure. (**A**) A buttonhole in the bleb was found in the eyes treated with prostaglandin. (**B**) Valve exposure developed in one eye that had been treated with aqueous suppressant and in two eyes that had been treated with prostaglandin analogues.

Valve exposure developed in two eyes of the PG analogue group and in one eye of the aqueous humour suppressant group but in no eyes of the control group (Fig. 8).

## Discussion

In this study, aqueous suppressants decreased the expression of α-SMA antibody, indicating the transformation of fibroblasts to myofibroblasts^21^, and collagen synthesis in the capsule after glaucoma shunt implantation. PG analogues upregulated IL-2 in the virgin Tenon’s tissue. Following tube surgery, PG analogues did not affect collagen deposition in the bleb. Four weeks after glaucoma implant surgery, IOP was lower in the aqueous suppressant group than in the control or PG group.

In the non-operated eyes, cytokines were evaluated in the aqueous humour as well as in the Tenon’s tissue. In this study, cytokines related to fibrosis or inflammation in the aqueous humour were not affected by treatment with aqueous humour suppressants or PG analogues. Lower expression of α-SMA and collagen in response to aqueous suppressants might not be caused by alterations in cytokines in the aqueous humour.

MMP-1, -3, and -9 levels were found to be upregulated by topical PG analogues in the conjunctiva and in the *in vitro* study using Tenon’s fibroblasts^22–24^. In this study, MMP-9 was upregulated in the PG group compared to the control group, but there was no significant difference between the groups. Short-term treatment of PG analogues might not induce a significant elevation in MMP levels in Tenon’s tissue.

In non-operated eyes, neither the aqueous suppressant nor the PG analogues significantly lowered the IOP below that of the control group (P > 0.05). The effects of aqueous suppressant eye drops on IOP reduction have been controversial in normotensive rabbits and can vary depending on the method of IOP measurement^25–29^, even though the effects of IOP reduction were potent in ocular hypertensive rabbits^30^. Topical application of PG analogues did not lower IOP significantly in the normotensive rabbits^31–33^. In the operated eyes, on the other hand, postoperative IOP was lower in the aqueous suppressant-treated eyes than in the control or PG-treated eyes (P < 0.05). Postoperative IOP reduction seemed to mainly result from the effects of the wound healing modulation induced by the aqueous suppressants because the IOP-lowering effect of the aqueous suppressant itself was not significant in the non-operated eyes. IOP was measured under general anaesthesia. Tiletamine mixed with zolazepam did not have a significant effect on IOP in cats or dogs^34,35^, whereas Xylazine, especially at high doses, decreased IOP in rabbits^36,37^. Given those findings, the actual IOP might be similar to or greater than the measured IOP in this study. We could not draw a conclusion based on IOP measurements alone. Therefore, we also analysed the cytokines in the aqueous humour and the Tenon’s tissue sample and visualized the histopathology of the blebs in this animal study.

We speculate that the mechanisms of the anti-fibrotic effects of aqueous suppressants on the wound healing response are as follows: first, aqueous humour contains various growth factors including TGF-β, which is a critical molecule for inducing fibroblasts to synthesise collagen and in their transformation to myofibroblasts^11^. Therefore, the suppression of aqueous humour flow might decrease the effects of aqueous humour during tube surgery. Second, aqueous humour encounters considerable resistance at the bleb; therefore, fibroblasts in the bleb might be affected by substantial pressure. The mechanical application of heavy pressure on fibroblasts has been found to stimulate fibroblast activation^38,39^. Therefore, aqueous suppressants might reduce fibrosis of the capsule through the quantitative attenuation of growth factors and the mechanical decrease of aqueous pressure to the bleb.

For PG analogues, the mean length of α-SMA was comparable to that of the control group at postoperative 4 weeks. Collagen deposition was not altered by treatment with PG analogues following glaucoma implant surgery. In human Tenon’s tissue, latanoprost stimulated collagen gel contraction but had no effects on collagen degradation by human Tenon’s fibroblast, corresponding to the findings of our animal study^17^. In the PG group, no alterations in collagen deposition in the bleb might result in similar postoperative IOP to that of the control group.

The PG group showed a relatively high rate of valve exposure. PG analogues stimulated the level of IL-2 in virgin Tenon’s tissue. IL-2 is one of the cytokines found in patients with intraocular inflammation^40^. PGF_2α_ has been reported to be involved in acute and chronic inflammatory disease^16^. The application of a non-steroidal anti-inflammatory drug attenuated the IOP-lowering effects of PG analogue anti-glaucoma eye drops in both rabbits and humans^41–43^. The association of PG analogues with inflammation might be related to the relatively high rate of valve exposure.

Short postoperative follow-up of the eyes is one of limitations of this study because collagen maturation starts at 1 week postinjury and continues for anywhere from 12 to 18 months in the wound healing response^11^. On average, the postoperative hypertensive phase develops at postoperative 1 month within the first 3 months of wound healing following GDD surgery^3^. Compared to humans, rabbits live short (8–10 years) and accelerated lives. Inflammatory responses, which are complex pathobiological processes, can be faster in the rabbits than in humans, depending on the phase of life^44^. Postoperative 4 weeks in rabbits might represent a longer postoperative period than the same time when applied to humans. In this study, the cytokines were analysed in the non-operated tissue because we speculated that it is difficult to differentiate the change in cytokines related to glaucoma medication in the wound healing response, in which various cytokines are involved. In the operated eyes, we focused on the histopathology of blebs associated with fibrosis. We did not use the experimental glaucoma model in this study, and this might be a limitation of our study. In glaucoma, the aqueous humour has higher concentrations of TGF-β and interleukin (IL)-8^45,46^. The effects of aqueous humour suppressants on wound healing after tube surgery might be greater in the experimental glaucoma model than in the non-glaucoma model because the glaucoma model might have higher TGF-β or inflammatory cytokine levels. The small sample size of each group is another limitation of this study. Both eyes were used in this study to decrease inter-individual differences and the total number of animals used in the study. The instillation of topical timolol in one eye can reduce IOP in the contralateral eye, although the amount of IOP reduction is usually about one half of that in the treated eye^47,48^, and this might be a limitation of our study. Following GDD surgery, steroid eye drops are usually used postoperatively. Corticosteroids have been found to inhibit cell attachment and proliferation in tissue culture studies of human Tenon capsule fibroblasts^49,50^. Further studies are needed to elucidate the effects of aqueous suppressant or prostaglandins after Ahmed glaucoma surgery when steroid is concomitantly used.

In summary, we demonstrated that early treatment with aqueous suppressant decreased fibrosis of the bleb after glaucoma shunt operation and lowered IOP at postoperative 1 month. PG treatment did not significantly affect collagen deposition in the bleb or postoperative IOP. When clinically selecting glaucoma eye drops after tube surgery, the effects of glaucoma medication on the wound healing response should be considered.

## Supplementary information

Supplementary file
