# Supplementary material for: Effects of aqueous suppressants and prostaglandin analogues on early wound healing after glaucoma implant surgery
Source: Sci Rep. 2019 Mar 27;9:5251. doi: 10.1038/s41598-019-41790-1 (PMC6437192; doi:10.1038/s41598-019-41790-1)
Supplement: Supplementary file 1 — Supplementary file [file 41598_2019_41790_MOESM1_ESM.pdf]

Effects of aqueous suppressants and prostaglandin analogues on early wound healing after glaucoma implant surgery

Kyoung In Jung, M.D., Ph.D., Jung Eun Woo, Chan Kee Park, M.D., Ph.D

-Supplementary files-

Supplementary table S1

Supplementary table S2

Supplementary table S3

Supplementary table S4

Supplementary table S5

Supplementary table S6

Supplementary table S1. Intraocular pressure in non-operated eyes and in operated eyes

		**Control**	**Aqueous suppressant**	**Prostaglandin analogue**	P value*
Non-operated eyes	Before treatment	8.7±1.5	8.6±1.7	9.1±1.2	0.670
	After treatment	8.7±1.5	8.0±1.8	8.9±0.5	0.545
Glaucoma drainage device implantation	Preoperative	9.1±1.7	8.6±1.7	9.2±1.2	0.727
	Postoperative 2 weeks	6.7±1.9	6.5±2.2	8.6±1.4	0.200
	Postoperative 4 weeks	9.9±1.3	5.9±0.9	10.7±1.6	**0.001**

Data are expressed as the means ± SD.

* Statistically significant differences (P<0.05) assessed by an ANOVA test

Supplementary table S2. Cytokine levels in aqueous humor in non-operated eyes

Cytokine (pg/ml)	**Control**	**Aqueous suppressant**	**Prostaglandin analogue**	P value
TGF β-1	8.1±5.1	5.2±4.6	3.2±5.0	0.296
TGF β-2	648.7±201.4	571.5±267.2	637.9±286.2	0.861
TGF β-3	12.5±5.2	8.3±2.8	9.4±5.1	0.317
IL-2	15.9±11.9	10.3±5.3	8.3±0.4	0.224
IL-6	1.9±0.1	1.9±0.2	2.1±0.1	0.055
MCP1	22.6±2.5	23.2±3.5	23.2±3.9	0.949
VEGF	14.5±5.3	16.2±3.3	12.3±2.0	0.217

Data are expressed as the means ± SD.

IL, interleukin; MCP, macrophage chemotactic protein; TGF, Transforming growth factor; VEGF, vascular endothelial growth factor

* Statistically significant d*iff*erences ($P<0.05$) assessed by an ANOVA test

Supplementary table S3. Cytokine levels of Tenon's tissue in non-operated eyes.

Cytokine (pg/ml)	**Control**	**Aqueous suppressant**	**Prostaglandin analogue**	P value	Post hoc
TGF β-1	66.5±26.0	34.2±26.2	93.7±58.5	0.063	
TGF β-2	173.0±163.1	129.2±124.6	101.6±55.2	0.607	
TGF β-3	7.2±6.7	4.3±5.5	3.4±4.1	0.483	
IL-2	18.4±15.4	31.9±22.6	57.8±15.0	**0.006**	Control or Aqueous suppressant <Travoprost
IL-6	2.7±0.5	2.7±0.3	2.5±0.4	0.654	
MCP1	33.6±4.6	33.4±1.6	28.9±4.0	0.066	
VEGF	20.7±11.0	18.5±8.1	19.0±9.3	0.916	
MMP3	4.7±1.7	5.1±2.0	4.9±1.5	0.922	
MMP9	21.6±9.7	21.7±8.5	26.0±9.2	0.677	
TIMP2	380.8±184.6	442.1±106.0	450.2±141.4	0.679	
TIMP4	23.3±1.8	24.3±2.9	24.7±3.2	0.657	

Data are expressed as the means ± SD.

IL, interleukin; MCP, macrophage chemotactic protein; MMP, matrix metalloproteinase; TGF, Transforming growth factor; TIMP, tissue inhibitor of metalloproteinase; VEGF, vascular endothelial growth factor

* Statistically significant d*iff*erences (P<0.05) assessed by an ANOVA test

Supplementary table S4. Histologic analysis of capsules surrounding the endplate using haematoxylin and eosin (H&E) staining.

		**Control**	**Aqueous suppressant**	**Prostaglandin analogue**	P value
Cellularity (number/mm^2)	Postoperative 2 weeks	6670.0±2094.3	5568.6±1914.6	6150.0±1143.7	0.556
	Postoperative 4 weeks	3690.0±472.5	3710.0±619.9	4044.3±813.1	0.631
Number of foreign body giant cells per slide	Postoperative 2 weeks	1.0±0.9	0.9±1.1	0.3±0.5	0.254
	Postoperative 4 weeks	0.5±0.5	1.0±1.7	0.9±0.3	0.716

Data are expressed as the means ± SD.

* Statistically significant d*iff*erences (P<0.05) assessed by an ANOVA test

Supplementary table S5. Mean height of the inner fibrous capsule stained by Masson trichrome staining at postoperative 4 weeks

	**Control**	**Aqueous suppressant**	**Prostaglandin analogue**	P value	Post hoc
Mean height of the inner fibrous capsule (μm)	364.0±74.6	282.7±34.0	382.1±42.2	0.013	Aqueous suppressant< Control or Travoprost

Data are expressed as the means ± SD.

* Statistically significant d*iff*erences (P<0.05) assessed by an ANOVA test

Supplementary table S6. Analysis of anti α-smooth muscle actin (SMA) immunohistochemical staining in the bleb

Mean length of anti-anti α-smooth muscle actin (SMA) positive bleb (μm)	Control	Aqueous suppressant	Prostaglandin analogue	P value	Post hoc
Postoperative 2 weeks	316.9±63.9	359.4±71.6	370.6±87.5	0.100	
Postoperative 4 weeks	267.6±64.3	220.9±28.2	274.3±31.7	**0.001**	Aqueous suppressant < Control or prostaglandin analogue

Data are expressed as the means ± SD.

* Statistically significant differences ($P<0.05$) assessed by an ANOVA test
